# Cytokine Biomarkers Associated with Human Extra-Pulmonary Tuberculosis Clinical Strains and Symptoms

**DOI:** 10.3389/fmicb.2018.00275

**Published:** 2018-02-21

**Authors:** Paulo Ranaivomanana, Mihaja Raberahona, Sedera Rabarioelina, Ysé Borella, Alice Machado, Mamy J. De Dieu Randria, Rivo A. Rakotoarivelo, Voahangy Rasolofo, Niaina Rakotosamimanana

**Affiliations:** ^1^Unité des Mycobactéries, Institut Pasteur de Madagascar, Antananarivo, Madagascar; ^2^Infectious Diseases, Joseph Raseta Befelatanana University Hospital, Antananarivo, Madagascar; ^3^Faculté de Médecine, University of Fianarantsoa, Fianarantsoa, Madagascar

**Keywords:** *Mycobacterium tuberculosis*, extra-pulmonary tuberculosis, immune response, cytokine, biomarker

## Abstract

**Background:** The primary site of infection for *Mycobacterium tuberculosis* (*Mtb*) is the alveolar macrophages. However, *Mtb* can disseminate into other organs and causes extrapulmonary tuberculosis (EPTB). The diagnosis of EPTB is challenging due to relatively inaccessible infectious sites that may be paucibacillary and with clinical symptoms varying by site that are similar to those seen in other diseases. Hence, we sought to identify the expression patterns of a variety of cytokines that may be specific to EPTB from *in vitro* infections and in the plasma of TB patients.

**Methods:** To define those cytokine secretions associated with EPTB, human THP-1 derived macrophages were first infected with *Mtb* clinical isolates from pulmonary and EPTB. Infected macrophages supernatants were harvested at different time points and cytokines known to play key roles in TB immune responses including TNF-α, IL-6, IL-10, IFN-γ, and VEGF-A were measured by ELISA. Those cytokines that were *in vitro* associated to EPTB were also measured in the plasma from patients with PTB, EPTB, non-EPTB-confirmed-like symptoms and healthy controls.

**Results:** While all of the studied cytokine secretions varied after *in vitro* infection, higher levels of TNF-α and VEGF secretions were observed *in vitro* in the infected macrophages respectively in the PTB and EPTB infecting clinical isolates. Similar trends were observed from the plasma of patients where patients with PTB showed significantly higher level of TNF-α compared to EPTB and healthy control groups. The patients with EPTB showed higher plasma level of VEGF compared to those patients with the non-EPTB (*p* < 0.01) and to healthy controls group (*p* < 0.0001). Using Receiver Operating Curves (ROC), we showed that TNF-α and VEGF concentrations could distinguish EPTB from non-confirmed EPTB with high sensitivity and specificity.

**Conclusion:** Pulmonary and extrapulmonary *Mtb* clinical isolates showed different cytokine induction pattern in human macrophages that is also found in the plasma level of the EPTB patients. Further investigations are needed to define cytokine secretions that can lead to the definition of bio-signatures to differentiate EPTB from other pathologies with confusing symptoms that hampered the diagnosis of TB.

## Introduction

With 10.4 million new cases and 1.5 million deaths in 2015 (World Health Organization [WHO], 2016), tuberculosis (TB) remains a major global public health problem. TB, an air transmitted infectious disease, is known to affect primarily the lungs; however, TB has also been described in virtually all tissues or organs (Alvarez and McCabe, 1984; Mazza-Stalder et al., 2012). Extrapulmonary TB (EPTB) represents about 20% of all TB cases in developing countries (Cagatay et al., 2004; Sharma and Mohan, 2004) and can be much higher in immune-compromised individuals. The diagnosis of EPTB is challenging due to the poor performance of conventional diagnostic techniques. Moreover, while the TB symptoms are constituted by local pain, weight loss, night sweat and fever (Golden and Vikram, 2005), there is no EPTB specific symptoms in any tissue or organ and the observed symptoms can be confused with those of other pathologies. This would further result in delayed diagnosis and treatment that can quickly lead to death depending on the severity of the affection (World Health Organization [WHO], 2016). Finding biomarkers to improve EPTB diagnosis can thus be important in the global control of TB.

The mechanism of *Mtb* dissemination from the pulmonary site to other organs is not well elucidated. Following entry of the bacillus in the lungs, alveolar macrophages invade the subtending epithelial layer and secrete several cytokines including the Th1 profile. These cytokines allow the recruitment and activation of inflammatory cells to form the granuloma that contains the pathogen (Orme and Basaraba, 2014; Gideon et al., 2015). The outcome of the infection will then depend on the imbalance of interactions between the host immune system response and the infecting bacteria (Ernst, 2012). Lymphohematogenous dissemination of *Mtb* is one of the key events in TB pathogenesis since it is involved in the development of protective T-cell mediated immune response but it also enables the bacteria to spread to new niches and therefore to establish alternative sites of infection (Krishnan et al., 2010). Genetic and host immune factors are suspected to be involved in extrapulmonary dissemination of *Mtb* and TB pathogenesis (Caws et al., 2008).

It was reported that the production of cytokines was different in persons healed from pulmonary TB when compared to those with EPTB (Hasan et al., 2009; Fiske et al., 2012). As development of EPTB seems to be the result of an immune host defects (Fiske et al., 2012), the discovery of factors that are associated to extrapulmonary disease will advance TB prevention efforts by identifying immune responses that could be boosted by TB vaccines.

The human host immune response against *Mtb* was shown to vary according to the genotype families of the infecting *Mtb* (Rakotosamimanana et al., 2010; Portevin et al., 2011) and studies using animal models identified some bacterial factors that were associated with extrapulmonary dissemination or colonization of specific organs (Pethe et al., 2001; Chawla et al., 2012). In a recent study using *Mtb* infected-macrophages and observations from TB patients, a correlation was found between *Mtb* infection and the production of angiogenesis factors (VEGF: Vascular endothelial growth factor) and subsequent vascularization during the bacterial dissemination into other organs (Polena et al., 2016). VEGF is a known major player in angiogenesis and lymphangiogenesis and is induced in response to tissue inflammation, hypoxia and pro-inflammatory cytokines (Ferrara et al., 2003; Koch et al., 2011).

Previous studied have reported cytokines TNF-α, IL-6, IL-1β, IL-10, IFN-γ, TGF-β and chemokine VEGF, levels to be significantly different in macrophage cells infected by mycobacteria compared to uninfected controls as well as in TB patients compared to healthy controls (Dlugovitzky et al., 1997; Morosini et al., 2003; Vankayalapati et al., 2003; Sahiratmadja et al., 2007; Dubois-Colas et al., 2014; Helguera-Repetto et al., 2014; Sousa-Vasconcelos Pda et al., 2015). However, there are few comparative studies describing the levels of cytokines and chemokines in patients with different clinical sites of TB, (PTB, EPTB) and EPTB-like pathologies. Moreover, studies which have investigated immune responses against EPTB have often combined patients without differentiating *Mtb* strains (Sharma et al., 2002; Jamil et al., 2007). It is important to consider the diversity associated with virulence capacity of these clinical isolates for inducing TB active disease and to evaluate the TB clinical forms (PTB or EPTB) and the immune response variations in relation to this bacterial diversity.

We propose here to identify human immune host signatures that could be involved in the dissemination of the pathogen and the colonization of other organs than the pulmonary sites that can be used for EPTB diagnosis by considering the genotype of infecting *Mtb*. In this study, we first determined the variations of cytokines TNF-α, IL-6, IL-10, IFN-γ and VEGF, in macrophages infected with different *Mtb* clinical isolates from patients with PTB and EPTB with *Mtb* strains belonging to the same Lineage 1 genotype known to enhance an elevated human pro-inflammatory responses (Rakotosamimanana et al., 2010; Portevin et al., 2011). Secondly, the variations of those cytokines productions presenting an *in vitro* difference between PTB and EPTB were measured in plasmas of patients and healthy controls to study their ability to distinguish the clinical groups of confirmed EPTB, EPTB-like symptoms and PTB patients.

## Materials and Methods

### Ethics Statement

The study was approved by the National Ethics Committee of the Ministry of Health of Madagascar (N°072-MSANP/CE of 14/08/2014). All the patients that participated in the *ex vivo* part of the study gave their informed consents before any process was performed.

### *Mtb* Clinical Isolates Selection and Mycobacteriology Procedures

The *Mtb* strains with EAI8_MDG spoligotype (shared-type 109) from the IPM-National Reference center for Mycobacteria clinical strains collection were used for the macrophage infections. PTB and EPTB isolated Mtb strains were matched by the same spoligotype, the gender/age/matched-patients. During the *in vitro* study, *Mtb* isolates from 7 different PTB and 7 other EPTB patients were used to *in vitro* infect the macrophages. Supplementary Table S1 described the characteristic of those clinical isolates and the TB patients they were isolated from. The clinical sites where the EPTB isolates came were heterogeneous: lymph node (*n* = 1), pleural (*n* = 2), cerebrospinal (*n* = 1), urine (*n* = 2), pus (*n* = 1). *Mtb* was grown in Middlebrook7H9 broth supplemented with albumin-dextrose-catalase (M0178 Middlebrook 7H9 Broth Base).

### Macrophages *in Vitro* Infection

The human monocyte–macrophage cell line THP-1 (Sigma) was maintained in culture medium containing RPMI-1640 medium supplemented with 200 mM L-glutamine, 10% inactivated foetal bovine serum(FBS), antibiotics (penicillin and streptomycin), and antifungal (fungizone) at 37°C, in a humidified 5% CO2 environment. The day before infection experiment, 100 nM phorbolmyristate acetate (Sigma) was added to differentiate cells into macrophages according to Spano et al. (2013) and distributed in 6-well plates (5 × 10^5^ cells /well). The cells were cultured in RPMI-1640 medium, with 10% inactivated FBS and 200 mM L-glutamine, without antibiotics or antifungal.

THP1-derived macrophages were infected as previously described (Tailleux et al., 2003) at a multiplicity of infection of 1/1 bacteria/cell. Briefly, before infection, mid-log phase *Mtb* strains were washed two times with PBS, clumps were disassociated by 100 passages through a needle, followed by 5 min of sedimentation. The density of bacteria in the supernatant was checked at OD 600 nm and correlated to the numeration of the aliquot to allow 1-to-1 bacterium-per-cell infections. The infection was performed in a six-well plate with 5.10^5^ cells/5.10^5^ bacteria/well in 2 ml complete medium (without antibiotics). Non-infected cells were used as control for the experiments. All experiments were performed in duplicate. After 3 h of incubation at 37°C and 5% CO_2_, infected cells were washed three times to remove extracellular bacteria and were incubated in fresh complete medium. Supernatants from control uninfected cells and *Mtb*–infected macrophages were harvested after 0 h (just after wash: 3 h post infection), 24, 48, and 120 h of macrophage infection, filtered using Millipore filter of 0.22 μm; and stored at -80°C for cytokine analysis.

Enumeration of intracellular bacteria was performed at 0 h (3 h post infection), 24, 48, and 120 h post-infection. Cell supernatant was removed and the wells were washed two times to remove extracellular bacteria and then lysed by cold distilled water with 0.05% Tween 20%. Cell lysates were diluted in 4 different dilutions and plated in triplicate on 7H11 solid medium complemented with OADC and incubated at 37°C. CFUs were scored after 3 weeks. CFUs were enumerated as previously described (Tailleux et al., 2003). All of the Mtb preparations and infections procedures were performed in a biosafety level 3 facility.

### Study Population and TB Diagnostic Tests

All patients with suspected TB present or referred at the Infectious Diseases Unit of the Joseph Raseta Befelatanana Hospital (HUJRB), the EUSSPA/DAT (Etablissement Universitaire de Soins et de Santé Publique Analakely/Dispensaire antituberculeux) and at the IPM’s anti-rabies center (healthy controls) were included in this study. Blood and specimens were harvested from the patients and plasma obtained through centrifugation. For suspected extrapulmonary TB patients, all clinical specimens (cerebrospinal fluid, pleural fluid, lymph node, ascitic fluid, pus) were considered and investigated for TB diagnosis. For PTB patients, sputum smear direct microscopy and LJ culture were performed. For all biological fluids samples for TB diagnosis by bacteriological detection of Mtb were beforehand decontaminated using the sodium lauryl-sulfate method. Then, one drop of previously decontaminated specimen was fixed on a slide, stained with auramine staining and examined under fluorescent microscopy. The remaining sample was cultured on standard Löwenstein-Jensen (LJ) medium.

The clinical criteria were: (1) EPTB: patients presenting suspected clinical symptoms of extrapulmonary TB regardless of the anatomic localization with positive smear acid-fast bacilli or *Mtb* positive on LJ culture; HIV positive patients and pregnant women were excluded from the study; (2) PTB: new TB cases with sputum smear AFB+ or *Mtb* positive on LJ culture; (3) Healthy control: healthy individual without any active TB symptoms after clinical investigation and chest X-ray, (4) non-EPTB: suspected-EPTB patients with negative *Mtb* in biological specimen culture.

### Cytokine Quantification by Enzyme-Linked Immunosorbent Assay, ELISA

After the *in vitro* infections, the concentrations of IL-10, IL-6, TNF-α, IFN-γ and VEGF-A in the thawed supernatant were determined by ELISA as described by the assay kit manufacturers (Duoset kit, R&D Systems). The concentrations of cytokines in the plasma from the patients were measured in duplicate by specific sandwich ELISA as described by the manufacturers (R&D Systems).

### Statistical Analysis

The normality of the each measures obtained was assessed with the D’Agostino & Pearson omnibus normality test. Multiple comparisons were performed using a two-way ANOVA adjusted by the Holm–Sidak post-test to assess the difference of CFU and cytokine concentrations according to the *in vitro* infection time points and the clinical groups. The one-way ANOVA test was used with multiple comparisons adjusted by the Tukey or the Dunn’s post-tests respectively for parametric and non-parametric tests to assess the plasmatic cytokine concentrations differences amongst the clinical groups. Receiver Operating Characteristic (ROC) analysis was used to assess the diagnostic strength of the plasmatic concentration of cytokines to distinguish the clinical groups. Results were considered significant if the 95% confidence interval (CI) of the area under the curve (AUC) exceeded 0.70. A p value below 0.05 was considered as significant. The Prism GraphPad6 Software was used for the statistical analysis.

## Results

### Similar Intra Macrophagic CFU Counts after *in Vitro* Infection with Isolates from Different Clinical Infectious Sites

**FIGURE 1:**
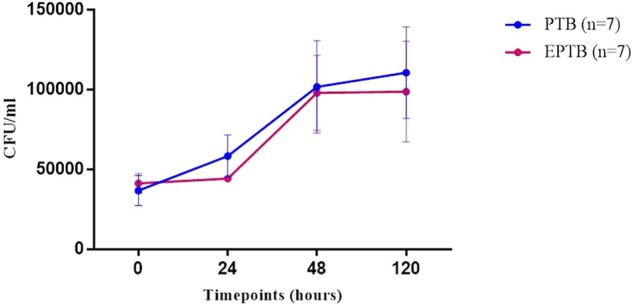
Bacterial growth curve showing the intracellular CFU of *Mtb* in the infected macrophages at different timepoints post infection (hours) with EPTB-isolated strains (red line, *n* = 7) and PTB-isolated strains (blue line, *n* = 7). CFU, colony forming unit; PTB, pulmonary tuberculosis strains; EPTB, extrapulmonary tuberculosis strain; ctrl, non-infected cells. Each point corresponds to the average of three determinations ± SD. Statistical comparison was performed with a two-way ANOVA (*p* = ns).

The enumeration of intracellular bacteria was performed at 0 h (3 h post infection), 24, 48, and 120 h post-macrophage infection. Infecting *Mtb* strains isolated from PTB and EPTB patients showed similar growth rates in the macrophages from t0 to t120 post-infection (**Figure 1**). No significant difference was observed in bacterial count at any post infection time points between PTB and EPTB strains.

### Different Level of Cytokine Secretions Depending on the Clinical Infectious Sites Origin of the Infecting Strains

**FIGURE 2:**
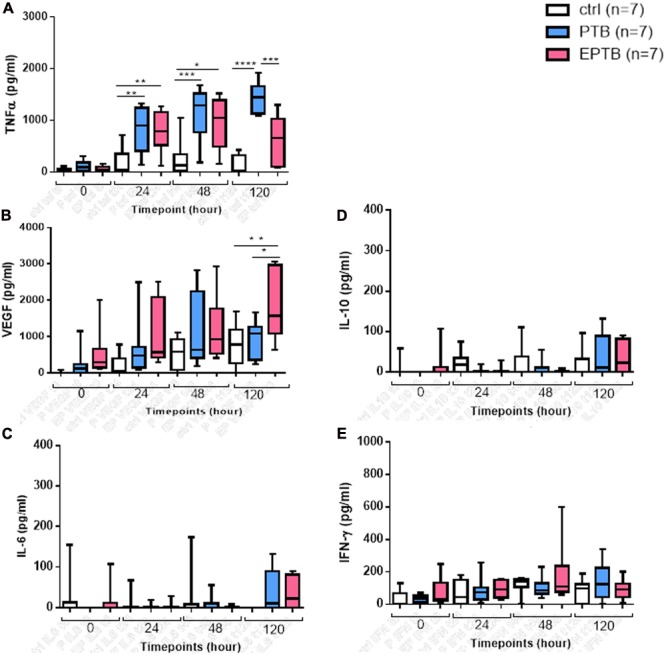
Cytokine secretions by THP-1 derived macrophages infected with *Mtb* strains. **(A)** TNF-α, **(B)** VEGF, **(C)** IL-6, **(D)** IL-10, **(E)** IFN-γ concentrations. Multiple comparisons were assessed with a two-way ANOVA for the different timepoints and the clinical group with a Holm–Sidak post-test. ^∗^*P* < 0.05, ^∗∗^*P* < 0.01. PTB, pulmonary tuberculosis strains; EPTB, extrapulmonary tuberculosis strain; ctrl, non-infected cells.

After quantifying the concentration of the cytokines harvested from the infected macrophages supernatants, trends of increased productions of all the studied cytokines were observed in comparison to uninfected macrophages (**Figure 2**). Despite the observation from the other cytokines production, the secretions of both the TNF-α and VEGF trends increased over time (**Figures 2A,B**) after 24, 48, and 120 h post-infection. Surprisingly, unlike the others cytokines, a slight increase of the VEGF secretion was observed within the uninfected macrophages. The release of this chemokine by the control cells also increased over time with a higher level at 120 h post infection. We next compared the cytokine responses between PTB and EPTB infecting isolate strains at the different time points. Tendency in differences in the level of cytokines produced by the THP-1 derived macrophage depending on the clinical site origin of the *Mtb* infecting strains. Tendency of higher levels of TNF-α were observed when the PTB-isolated strains infected the macrophages compared to the EPTB-isolated strains infected macrophages depending on the infection time point. A statistically significant higher level of TNF-α was observed at 120 h (**Figure 2A**, *p* < 0.001). Unlike the TNF-α, after adjusting with the basal secretion observed in the uninfected macrophages, a trend of higher level of VEGF was observed with the EPTB-isolated strains compared to the PTB isolates at all time points, with a significant difference in the secretion levels after 120 h post infection (*p* = 0.04, **Figure 2B**).

### Plasma Levels of TNF-α and VEGF Varied Depending on the Clinical Symptoms of the Patients

We measured the level of both cytokines that differed *in vitro* between PTB and EPTB in the plasmas collected from patients with different clinical symptoms. Eighty six (86) persons were included in the study. Fourty-four participants were suspected of EPTB with quite heterogeneous symptoms and included a large variety of clinical specimens; the most frequent were cerebrospinal fluid (*n* = 18, 39.06%) and pleural fluid (*n* = 17, 28.12%). Finally, according to the bacteriology, EPTB was confirmed in 16 patients, 13 had confirmed PTB, 28 had EPTB-like symptoms without bacteriological confirmation and 29 were healthy controls. **Table 1** described the characteristic of those TB patients.

**Table 1:** Baseline characteristics of patients and type of specimen.

	EPTB	Non-EPTB	PTB	CTRL
	*N* = 16	*N* = 28	*N* = 13	*N* = 29
Gender^∗^: *n* (%)				
Female	10 (35.71)	10 (35.71)	4 (30.77)	15 (51.72)
Male	18 (64.29)	18 (64.29)	9 (69,23)	14 (48.28)
Age# : Mean (*SD*)	32 (12.18)	35 (15.92)	30 (7.76)	32 (10.62)
Sputum and blood harvested	Sputum/blood	Sputum/blood	Sputum/blood	blood
Specimen harvested: *n* (%)				
Pleural fluid	1 (6.25)	5 (17.86)		
Lymph nodes	3 (18.75)	2 (7.14)		
Ascitic fluid	1 (6.25)	10 (35.71)		
Cerebrospinal fluid	7 (43.75)	11 (39.26)		
Pus	1 (6.25)			
Miliary	3 (18.75)			

*^∗^A chi-square test to determine whether the gender distribution was equal in the clinical groups was not statistically significant χ^*2*^(4, *N* = 86) = 2.23, *p*-value < 0.527. ^#^A one-way ANOVA with multiple comparisons adjusted by the Tukey post-test performed to assess the age differences amongst the clinical groups was not statistically significant (*p* = 0.62).*

**FIGURE 3:**
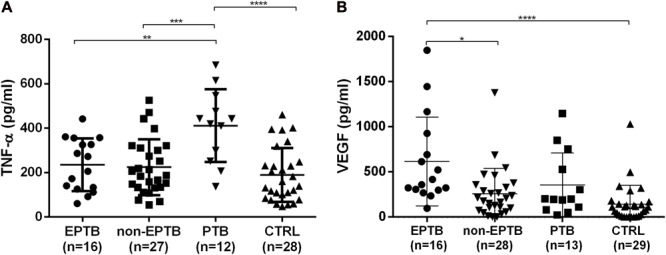
Quantity of TNF-α **(A)** and VEGF **(B)** secreted in the plasma of EPTB, non-EPTB, PTB, and healthy controls group. Multiple comparisons were assessed with a one-way ANOVA for two clinical groups adjusted with Tukey post-test **(A)** or Dunn’s post-test **(B)**: ^∗^ *P* < 0.05, ^∗∗^*P* < 0.01, ^∗∗∗^P < 0.001, ^∗∗∗∗^*P* < 0.0001. EPTB: bacteriologically confirmed extrapulmonary tuberculosis patients; non-EPTB, suspected-EPTB patients not bacteriologically confirmed; PTB, pulmonary tuberculosis patients; CTRL, healthy control group.

After comparing the plasma level of the two different cytokines in the four clinical groups, PTB patients showed a significantly higher production of TNF-α compared to the EPTB patients, to the EPTB-like and to CTRL group, (*p* = 0.004, *p* < 0.001, and *p* < 0.0001 respectively) (**Figure 3A**). Differently from the observations in the *in vitro* infections, the plasma of EPTB patients showed a higher concentration of VEGF to the non-EPTB (*p* = 0.022) and to healthy controls group (*p* < 0.0001). No statistical differences were observed when comparing plasma level of VEGF in EPTB patients to PTB (**Figure 3B**).

### Plasma Level of TNF-α and VEGF Concentrations Allowed to Respectively Distinguishing PTB and EPTB from the Other Patients Groups

**FIGURE 4:**
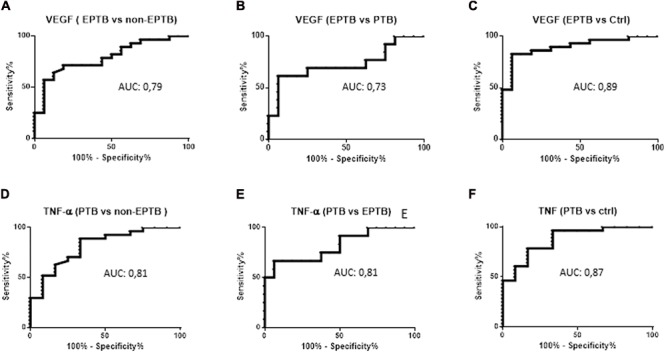
Receiver operating curves (ROC) analysis of TNF-α and VEGF as markers to distinguish the differen clinical groups. VEGF ROC between EPTB and non-EPTB **(A)**, PTB **(B)**, Ctrl **(C)**, TNF-α ROC between PTB and non-EPTB **(D)**, EPTB **(E)**, Ctrl **(F)**. EPTB, bacteriologically confirmed extrapulmonary tuberculosis patients; non-EPTB, suspected-EPTB patients not bacteriologically confirmed; PTB, pulmonary tuberculosis patients; CTRL, healthy control group.

We asked whether the plasmatic cytokines concentration could be useful for detecting a bacteriologically confirmed EPTB from the other clinical groups. Baseline levels of TNF-α and VEGF showed significant area under the curve (AUC) to respectively distinguish PTB and EPTB from the other clinical groups (**Tables 2, 3** and **Figure 4**). The TNF-α showed more than 80% AUC yielded when distinguishing PTB from the other clinical groups (*p* < 0.01) (**Table 3** and **Figures 4D–F**). Moreover, the plasmatic VEGF concentration yielded an AUC of 73% (*p* < 0.05) when compared to PTB (**Table 2** and **Figure 4B**), 79 and 89% (*p* < 0.01) in distinguishing bacteriologically confirmed EPTB respectively from the other pathology with extrapulmonary symptoms and healthy control group (**Table 2** and **Figures 4A,C**)

**Table 2:** Area-under-the-curve (AUC) of VEGF as to distinguish confirmed EPTB from the other clinical groups.

Host cytokine	Clinics	AUC (95% CI)	*p*-value	Sensitivity % (95% CI)	Specificity % (95% CI)
VEGF					
	Non-EPTB	0.79 (0.65–0.92)	0.0015	57 (37–75)	93 (69–99)
	PTB	0.73 (0.53–0.92)	0.0353	61 (31–86)	93 (69–99)
	CTRL	0.89 (0.80–0.99)	<0.0001	82 (64–94)	93 (69–99)

**Table 3:** Area-under-the-curve of TFN-α as to distinguish confirmed PTB from the other clinical groups.

Host cytokine	Clinics	AUC (95% CI)	*p*-value	Sensitivity % (95% CI)	Specificity % (95% CI)
TFN-α					
	Non-EPTB	0.81 (0.66–0.95)	0.002	51 (31–71)	91 (61–99)
	EPTB	0.81 (0.65–0.98)	0.0046	66 (34–90)	93 (69–99)
	CTRL	0.87 (0.75–0.99)	0.0002	60 (40–78)	91 (61–99)

## Discussions

The EPTB represents a non-negligible proportion of the TB active cases; however, its diagnosis that is lying firstly on clinical symptoms considerations is difficult due to the confusing symptoms with other pathologies as any human tissue or organ can be affected by EPTB while the traditional bacteriological tools to confirm TB were challenged by relatively inaccessible infectious sites that may be paucibacillary. In order to establish confirmation, invasive procedures are often necessary, making the diagnosis more difficult. The main objective of this study was to propose EPTB specific biomarkers deduced by the *in vitro* comparison of TB-associated cytokine response induced by different clinical isolates (PTB vs. EPTB) from human macrophages that were then assessed in human patients. Different cytokines known to be involved in the anti-tuberculosis immune response such as IFN-γ, TNF-α, IL-6, IL-10 and VEGF were first measured in human commercial cell-line derived infected macrophages to consider the host genetic effects on the immune response that was widely reported to be associated with EPTB (Caws et al., 2008). Although the THP-1 cell line was widely considered to be a good model of macrophages for *in vitro Mtb* infection studies (Riendeau and Kornfeld, 2003; Theus et al., 2005; Iona et al., 2012), it should not be forgotten that these are tumor cells and that certain functions remain poorly controlled. However, similar cytokine productions trends observed in this macrophage model were also observed *ex vivo* in human plasma in our study.

Moreover, the characterization of the determinants of *Mtb* virulence is an important process for understanding the pathogenesis of TB. The bacterial genetics effects on cytokine production were also considered here by infecting the macrophages with *Mtb* from similar bacterial lineages. Infecting the macrophages with the EAI-MDG8 (lineage 1) was preferred as the *Mtb*-lineage 1 strains were associated with an increased pro-inflammatory responses that would boost the cytokine levels we want to study (Rakotosamimanana et al., 2010; Portevin et al., 2011). Moreover, the *Mtb* EAI-MDG8 genotype was a predominant spoligotype profile in Madagascar where the study was performed (Rakotosamimanana et al., 2010).

The molecular and cellular mechanism of *Mtb* dissemination is not yet well understood. The cytokine network plays a central role in the inflammatory response and outcome of mycobacterial infections (van Crevel et al., 2002) and can be useful for a correct, specific and timely diagnosis is essential for EPTB. It has been reported that mycobacteria counteract the defense mechanisms deployed by the host immune system by altering the cytokine profile (van Crevel et al., 2002; Sousa-Vasconcelos Pda et al., 2015). TNF-α, IL-6, IL-10 IFN-γ, and VEGF play important roles in the immune response to the outcome of mycobacterial infections (van Crevel et al., 2002; Polena et al., 2016).

Our results indicate that EPTB strains elicit reduced TNF-α responses compared to the PTB strains indicating their ability to down-regulate one of the powerful pro-inflammatory response, which is crucial for controlling mycobacterial infections. The strongest evidence for the role of TNF-α to prevent TB pathogenesis was well reported in patients treated with TNF-α antagonist (Keane et al., 2001; Gardam et al., 2003; Askling et al., 2005; Wong et al., 2007a; Wallis, 2008; Martin-Mola and Balsa, 2009; Yasui, 2014). Moreover, it was shown that neutralization of TNF-α could induce dissemination of *Mtb* (Lin et al., 2010) but the mechanism was not well investigated. Previous study showed low TNF-α production by peripheral blood mononuclear cells from patients presenting EPTB but the mechanism has not been elucidated (Sterling et al., 2001; Fiske et al., 2012). The role of TNF-α in the control of bacilli in the latent stage has also been demonstrated by the reactivation of tuberculous infection (including miliary and extrapulmonary) in patients with Crohn’s disease and rheumatoid arthritis, after treatment with monoclonal anti-TNF-α antibodies (Keane et al., 2001). Similar results were also reported in a study using mice unable to synthesize TNF-α, which has increased susceptibility to TB (Bean et al., 1999). Thus, in the present study, the ability of EPTB-isolated strains to down regulate TNF-α production may be associated with a lower immune response and then a mechanism that would allow dissemination. It has been shown that the neutralization of TNF-α can induce the spread of *Mtb* (Mayordomo et al., 2002; Lin et al., 2010). Our results are comparable with those observed in another study showing a significant reduction in the production of TNF-α in (*ex vivo*) macrophages infected by hypervirulent strains of *Mtb* isolated from patients with tuberculous meningitis and where the production of IL-10 and IL-12 was even undetectable (Wong et al., 2007b). Despite a relatively high level of TNF-α observed in the healthy controls that may be due to latent *Mtb* infection or exposure due to the fact that the study was performed in a TB high incidence area, the difference between PTB and EPTB was also observed in the plasma of the patients from the present study. This cytokine acts in synergy with IFN-γ to increase the production of metabolites of nitric oxide and to eliminate mycobacteria and is essential for the formation of granulomas for the confinement of a mycobacterial infection (Gomez-Reino et al., 2003; Bottasso et al., 2007). While some studies identified the IFN-γ or the type I interferons as biomarkers for EPTB when compared to healthy controls that was also observed in the present study (Goyal et al., 2016), no difference was observed when comparing the PTB vs EPTB for the IFN-γ *in vitro* response that would probe for an host genetic effects associated with the IFN responses differences observed in EPTB.

During the *in vitro* macrophage infection, unlike TNF-α, our results indicated that VEGF production is significantly induced by the EPTB-isolated strains compared to PTB strains. This chemokine was also significantly higher in the plasma of EPTB-confirmed patients compared to patients with EPTB-like symptoms, but we did not observe a statistic difference with PTB patients. The VEGF is best known for its role as an activator of angiogenesis. In cancer, the angiogenesis induces tumor progression and metastasis (Carmeliet, 2005). Angiogenesis would play a similar role in TB and may well be involved in extrapulmonary forms of the disease. Indeed, a recent study argues in favor of this hypothesis. In zebrafish model, the formation of new blood vessels facilitates the dissemination of *M. Marinum* (Oehlers et al., 2015). Moreover, high VEGF levels were observed in tuberculous pleural effusion (Seiscento et al., 2010; Qama et al., 2012) and tuberculous meningitis (Matsuyama et al., 2001; van der Flier et al., 2004). Thus, other studies have used anti-VEGF agents in EPTB therapy to prevent bacteria from disseminating and inhibiting their growth (Invernizzi et al., 2015; Oehlers et al., 2015). Moreover, the results observed in the present study are in agreement with previous studies which have shown that the production of VEGF is positively stimulated in EPTB disease (Seiscento et al., 2010; Thayil et al., 2011; Misra et al., 2013; Zucchi et al., 2013). Similarly to the present study, Qama et al. (2012) reported an increase of both TNF-α and VEGF in EPTB patients compared to healthy controls from tuberculous pleural effusions with notably a 31-fold increase of the VEGF produced in the bronchoalveolar lavage fluids of patients with pleural TB compared to healthy control subjects (Qama et al., 2012). Thus, these observations confirm the crucial role of VEGF in the diffusion of *Mtb*. However, in other studies, VEGF was mostly considered as a biomarker of active PTB disease (Matsuyama et al., 2000; Abe et al., 2001; Alatas et al., 2004; Riou et al., 2012; Mihret et al., 2013; Ota et al., 2014). Further investigation is therefore needed to decipher the exact role of VEGF in TB, especially in its disseminated forms.

It is noteworthy to mention that our study has several limitations. First, despite our aim to propose tools that can be used in resources limited area the studied cytokine panel was only restricted to the well known TB-associated cytokines. The current observations could benefit from the proteomic or transcriptomic high-throughput tools that would target more and/or new cytokines (Blankley et al., 2016; Roe et al., 2016). Increasing the sample size would also strengthen the statistical differences observed to get a stronger distinction of EPTB with the other clinical symptoms depending on the cytokine quantification as well as getting enough biological materials to investigate the production of these cytokines in clinical sites other than blood. Moreover, as we were able to study the effects of the infecting strains during the *in vitro* infections, we did not study the genotypes of the strains infecting the confirmed EPTB patients even the cytokine variations were quite similar. Furthermore, despite the fact that we were able to distinguish the EPTB from the cytokine secretions, we did not obtained the final diagnostic of those suspected EPTB that were not confirmed bacteriologically that is cruelly lacking in most of low-resources countries. Finally, in future studies, it would be interesting to investigate other factors such as malnutrition (Edwards et al., 1971) or immunodeficiency (e.g., CD4 + depletion, Slutsker et al., 1993; Jones et al., 1997; Golden and Vikram, 2005) and other factors such as a history of renal insufficiency may be associated with an increased risk of TB and are therefore potential confounders for risk factors for EPTB (Gonzalez et al., 2003; Abdelrahman et al., 2006; Sen et al., 2008; Lin et al., 2009).

## Conclusion

This study showed that *Mtb* PTB and EPTB strains have different cytokine/chemokine profile induction in macrophages. EPTB strains are characterized by strong VEGF induction/low TNF-α induction and inversely with PTB strains. We conclude that this ability to reduce TNF-α production and increase VEGF secretion can be considered as a specific biomarker in EPTB disease. Specifically, in this study the up-regulation of VEGF secretion could help in evaluating suspected EPTB compared to other affections in the body. These findings can be useful for understanding the immune response to *Mtb* infection and help to understand factors that may influence the progression to extrapulmonary disease. Although the exact mechanism leading to these differences is still poorly understood and the panel of cytokines limited, our observations may serve as a basis for future studies. Finally, the mechanisms of induction or inhibition of the secretion of these cytokines must be well elucidated in order to better anticipate the fight against severe forms of TB in particular disseminated TB such as meningitis and miliary TB which are often fatal.

## Author Contributions

Substantial contributions to the conception or design of the work, or the acquisition, analysis, or interpretation of data for the work: NR, VR, PR, MR, RR, MJDR, SR, AM, and YB. Drafting the work or revising it critically for important intellectual content: NR, VR, PR, MR, RR, MJDR, SR, AM, and YB. Final approval of the version to be published: NR, VR, PR, MR, RR, MJDR, SR, AM, and YB. Agreement to be accountable for all aspects of the work in ensuring that questions related to the accuracy or integrity of any part of the work are appropriately investigated and resolved: NR, VR, PR, MR, RR, MJDR, SR, AM, and YB.

## Conflict of Interest Statement

The authors declare that the research was conducted in the absence of any commercial or financial relationships that could be construed as a potential conflict of interest.
