# Supplementary material for: Cytokine Biomarkers Associated with Human Extra-Pulmonary Tuberculosis Clinical Strains and Symptoms
Source: Front Microbiol. 2018 Feb 21;9:275. doi: 10.3389/fmicb.2018.00275 (PMC5826350; doi:10.3389/fmicb.2018.00275)
Supplement: Supplementary file 1 [file Table_1.docx]

**Table S1**: General characteristic of the patients where the clinical strains were isolated

|  | **EPTB** (n=7) | **PTB** (n=7) |
| --- | --- | --- |
| **Age**: mean (SD) | 25 (±26.33) | 25 (±22.94) |
| **Sex**: N (%) |  |  |
| Male | 3 (42.85) | 4 (57.15) |
| Female | 4 (57.15) | 3 (42.85) |
| **Specimens**: N (%) |  |  |
| Lymph node | 1 (14.28) | Sputum |
| Pleural | 2 (28.57) |  |
| Cerebrospinal | 1 (14.28) |  |
| Urine | 2 (28.57) |  |
| Pus | 1 (14.28) |  |
| **Strain genotype (**n=14) | EAI8_MDG | EAI8_MDG |
